# Predictors of plasma leakage among dengue patients in Thailand: A plasma-leak score analysis

**DOI:** 10.1371/journal.pone.0255358

**Published:** 2021-07-29

**Authors:** Sutopa Talukdar, Vipa Thanachartwet, Varunee Desakorn, Supat Chamnanchanunt, Duangjai Sahassananda, Mukda Vangveeravong, Siripen Kalayanarooj, Anan Wattanathum

**Affiliations:** 1 Faculty of Tropical Medicine, Department of Clinical Tropical Medicine, Mahidol University, Bangkok, Thailand; 2 Faculty of Tropical Medicine, Information Technology Unit, Mahidol University, Bangkok, Thailand; 3 Department of Medical Services, Queen Sirikit National Institute of Child Health, Ministry of Public Health, Bangkok, Thailand; 4 Department of Medicine, Pulmonary and Critical Care Division, Phramongkutklao Hospital, Bangkok, Thailand; Mayo Clinic Minnesota, UNITED STATES

## Abstract

Delayed plasma leakage recognition could lead to improper fluid administration resulting in dengue shock syndrome, subsequently, multi-organ failure, and death. This prospective observational study was conducted in Bangkok, Thailand, between March 2018 and February 2020 to determine predictors of plasma leakage and develop a plasma leakage predictive score among dengue patients aged ≥15 years. Of 667 confirmed dengue patients, 318 (47.7%) developed plasma leakage, and 349 (52.3%) had no plasma leakage. Multivariate analysis showed three independent factors associated with plasma leakage, including body mass index ≥25.0 kg/m^2^ (odds ratio [OR] = 1.784; 95% confidence interval [CI] = 1.040–3.057; P = 0.035), platelet count <100,000/mm^3^ on fever days 3 to 4 (OR = 2.151; 95% CI = 1.269–3.647; P = 0.004), and aspartate aminotransferase or alanine aminotransferase ≥100 U/l on fever days 3 to 4 (OR = 2.189; 95% CI = 1.231–3.891; P = 0.008). Because these three parameters had evidence of equality, each independent factor was weighted to give a score of 1 with a total plasma-leak score of 3. Higher scores were associated with increased plasma leakage occurrence, with ORs of 2.017 (95% CI = 1.052–3.869; P = 0.035) for score 1, 6.158 (95% CI = 2.914–13.015; P <0.001) for score 2, and 6.300 (95% CI = 2.419–16.407; P <0.001) for score 3. The area under the receiver operating characteristics curves for predicting plasma leakage was good (0.677 [95% CI = 0.616–0.739]). Patients with a plasma-leak score ≥1 had high sensitivity (88.8%), and those with a plasma-leak score of 3 had high specificity (93.4%) for plasma leakage occurrence. This simple and easily accessible clinical score might help physicians provide early and timely appropriate clinical dengue management in endemic areas.

## Introduction

Dengue caused by the dengue virus (DENV) and spread by *Aedes aegypti* mosquito is currently a global burden, and dengue cases have increased by 8 fold over the last 10 years, with 70% of the global burden from Asia [1]. The reports from the Bureau of Epidemiology of Thailand from 2001–2016 showed an annual dengue incidence of 62–238 cases per 100,000 population with indefinable trends. Approximately 60–70% of the patients were adults aged ≥15 years [2].

The case fatality rate decreased from 0.18 in 2001 to 0.10 in 2016, but the mortality rate was higher in adults aged >40 years, ranging from 0.19–0.30 [2]. A previous study showed dengue shock syndrome (DSS) as the most common cause of death in adults and children with dengue accounted for 73%, followed by severe organ involvement (69%) and severe bleeding (30%) [3]. Delayed plasma leakage recognition could lead to inappropriate fluid management, resulting in DSS and subsequent multi-organ failure and death [4–6]. In addition, a previous Brazilian study reported plasma leakage and organ failure as the main indications for dengue patients’ hospitalization, and there was an association between plasma leakage and dengue mortality [7].

The World Health Organization (WHO) has proposed timely and appropriate clinical management, which involves dengue diagnosis and intravenous rehydration as the strategy to reduce dengue mortality to almost zero [4]. Several studies have attempted to investigate plasma leakage predictors among dengue patients aged ≥15 years. However, studies reported on different parameters, including demographic characteristics of older age [8–11], gender [8,11], ethnicity [11], diabetes mellitus [9,10–12], hypertension [11], delayed hospitalization [9], secondary infection [9], clinical parameters of bleeding [8], abdominal pain [8,10], lethargy [8,9], or cough [8], and laboratory findings of hematocrit (HCT) rising [12–14], thrombocytopenia [13,15], abnormal coagulation profile [14], raised liver enzymes [12,13], low serum albumin (ALB) level [13,15], or thickening of the gall bladder wall [9]. Recent studies have added several new parameters, including procalcitonin [16], lactate [16,17], chymase [18], and cytokines [19], as plasma leakage predictors among dengue patients aged ≥15 years.

Some of these laboratory parameters may not be accessible in remote and resource-limited settings, where patients at risk for plasma leakage need to be identified, using simple clinical assessment methods and easily accessible laboratory investigations to improve healthcare utilization efficiency and save patients from unnecessary expenditure, loss of productivity, morbidity, and mortality associated with dengue. Furthermore, no study developed a simple clinical score to determine plasma leakage, which might help physicians provide timely and appropriate clinical management for dengue in endemic areas.

Thus, a prospective observational study was conducted at the Hospital for Tropical Diseases in Bangkok, Thailand, between March 2018 and February 2020 to determine predictors of plasma leakage and develop a predictive score for plasma leakage among dengue patients aged ≥15 years.

## Materials and methods

### Ethical considerations

The study was approved by the Ethics Committee of the Faculty of Tropical Medicine, Mahidol University in Bangkok, Thailand (TMEC 17–084). This study followed the Strengthening the Reporting of Observational Studies in Epidemiology (STROBE) statement [20] and the Standards for the Reporting of Diagnostic (STARD) accuracy [21]. All potential participants who visited the outpatient department (OPD) or inpatient department (IPD) for the management of dengue were invited to participate in the study. Before participation, voluntary written informed consent was obtained from all patients or the patient’s guardians if they were 15–18 years old.

### Study design and population

This was a prospective observational study conducted at the Hospital for Tropical Diseases, Faculty of Tropical Medicine, Mahidol University in Bangkok, Thailand, between March 2018 and February 2020. Patients who visited OPD or IPD for dengue management and met the study criteria were included. The inclusion criteria were aged ≥15 years, being clinical dengue patients, defined as acute fever <7 days and having ≥2 of the symptoms, including headache, retro-orbital pain, myalgia, arthralgia or bone pain, rash, bleeding, leukopenia defined as white blood cell count (WBC) ≤5.0 ×10^3^ cells/mm^3^, thrombocytopenia defined as platelet (PLT) count ≤150 ×10^3^/mm^3^ or HCT rising ≥5% with positive DENV NS1 and/or anti-DENV IgM. The real-time reverse transcriptase-polymerase chain reaction (rRT-PCR) or micro-neutralization test was used to confirm dengue in all potential patients enrolled for this study. Patients who were not followed-up and those with poor blood specimen quality or errors in the pre-analytical process were excluded from the study.

The baseline characteristics data, comorbid conditions, symptoms, signs, and laboratory investigations were collected at the start of management. Then, follow-up information data were collected daily from patients treated at both OPD and IPD. At a 2-week follow-up appointment, blood samples were collected for complete blood counts and DENV infection confirmation. All data were recorded in a pre-designed case record form. All patients were managed by their attending physicians according to the standard guidelines for dengue management [5,6]. Tests were performed to obtain data on routine monitoring parameters, including the day of fever, clinical condition, vital signs, and complete blood count, during the follow up of the patients. Other tests, for data on additional laboratory parameters including liver enzymes, serum ALB, and chest radiography, were performed according to the attending physicians’ instructions, as per the clinical condition of the patients. Urine output was recorded for patients treated at the IPD. Plasma leakage was summarized at discharge date and defined as a rise in HCT ≥20%, clinical fluid accumulation by detecting pleural effusion or ascites using physical examination or chest radiography, and/or hypoproteinemia determined by serum ALB ≤3.5 g/dl or decrease ≥0.5 g/dl below baseline [5].

### Real-time reverse-transcriptase polymerase chain reaction (rRT-PCR)

DENV RNA was detected from patient serum on the first day of presentation using a two-step PCR method, as described by Lanciotti *et al*. [22], and modified using the methods of Reynes *et al*. [23]. PureLink® Viral RNA/DNA Mini Kit (Invitrogen™, Grand Island, NY, USA) was used to detect Dengue RNA from acute serum samples according to the manufacturer’s instructions.

### Micro-neutralization test

Serum samples collected at presentation to hospital and two weeks after the first presentation were assayed for serotype-specific DENV using the micro-neutralization test described by Vorndam *et al*. [24], with the slightly modified protocol of Putnak *et al*. [25]. The micro-neutralization test based on the principle of the plaque reduction neutralization test was used to measure all four serotype-specific anti-DENV neutralizing antibodies. Serum samples were tested in triplicate and serially diluted by 2-fold from 1:20 to 1:5120 in a 96-well microplate. Each microplate had controls, including media only (negative control), virus control, and sera of known specific DENV serotypes (positive controls). The virus control with viral foci counts in the range of 50–60 foci per well and media only control with no foci were included. Compared to the virus control, sera of known DENV serotypes showing at least 50% viral replication inhibition was required (25–30 foci per well). The DENV neutralization titer was defined as the reciprocal of the serum dilution, demonstrating 50% inhibition of DENV replication compared with the DENV control. A positive serotype-specific anti-DENV test was defined as a 4-fold rise in neutralizing antibody titer in paired samples for DENV serotypes 1 to 4.

### Sample size calculation

A previous report showed a plasma leakage incidence of 36.4% dengue patients in Bangkok, Thailand [26]. Thus, a sample size of 640 patients was needed for this study, using an incidence rate of 36.4% for plasma leakage with an error allowance of 3.5% and estimating 15% loss to follow-up and inadequate leftover samples.

### Statistical analysis

All data were analyzed using SPSS software (version 18.0; SPSS Inc., Chicago, IL). Kolmogorov-Smirnov test was used to analyze for normal distribution of numerical variables. Variables with non-normal distribution were summarized as medians and interquartile ranges (IQRs) and compared using Mann-Whitney *U* tests. Categorical variables were expressed as frequencies and percentages and analyzed using chi-square or Fisher’s exact tests. A univariate logistic regression analysis was performed with each potential factor included as an independent variable and the presence or absence of plasma leakage as the dependent variable. Patients’ characteristics, clinical and laboratory findings associated with plasma leakage development were categorized. The categorical parameters that were early, clinically significant, and not subjective were evaluated.

Any variable with a P ≤0.2 was considered potentially significant and was further analyzed in a stepwise multivariate logistic regression analysis using a backward selection method for determining significant independent factors. The optimal cut-off values of the factors for the prediction of plasma leakage were determined using the area under the receiver operating characteristics (AUROC) curves. Prognostic parameters were evaluated using 2×2 tables, and 95% confidence intervals (CIs) were calculated to determine sensitivity, specificity, positive predictive value (PPV), negative predictive value (NPV), positive likelihood ratio (LR+), and negative likelihood ratio (LR–). The optimal cut-off values were then combined in a single “score,” as described by Gibot *et al*., 2012 [27]. As per the score, one point was attributed to each independent factor’s presence. The score was classified as 0 (absence of all independent factors), 1 (presence of one independent factor), 2 (presence of two independent factors), or 3 (presence of three independent factors). The score was then further tested by multivariate logistic regression analysis for predictive value in plasma leakage. All tests of significance were two-sided, with a P <0.05 indicating statistical significance.

## Results

### Study population

**Fig 1:**
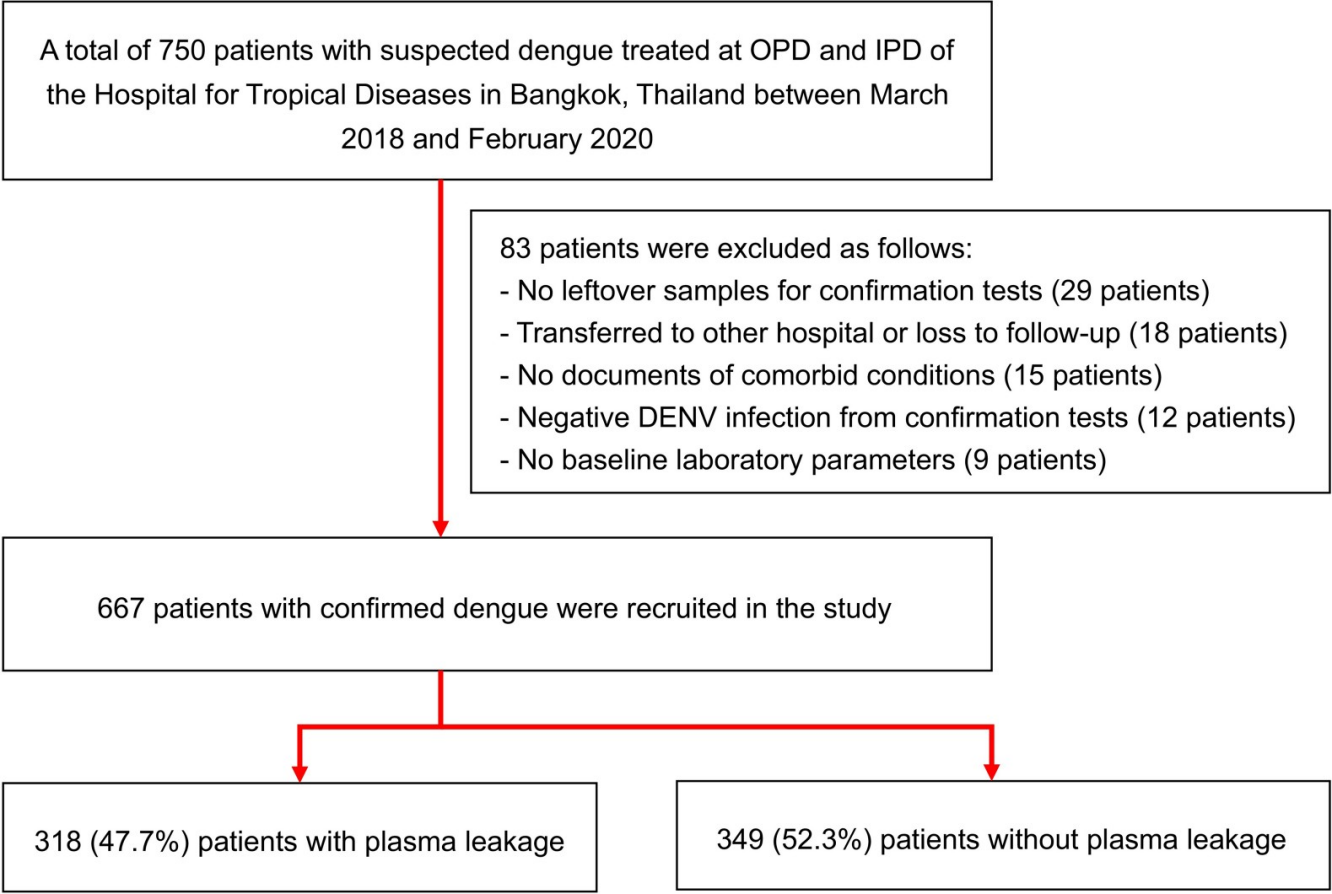
Flow diagram showing the enrollment of study patients. DENV, dengue virus; IPD, inpatient department; OPD, outpatient department.

A total of 750 suspected dengue patients visited the hospital during the study period. Of 750 suspected dengue patients, 83 were excluded due to lack of leftover DENV infection samples for a confirmation test (29 patients), transfer to other hospital or loss to follow-up (18 patients), no comorbid conditions documentation (15 patients), negative DENV infection confirmatory results, using rRT-PCR and micro-neutralization test (12 patients), and lack of baseline laboratory parameters (9 patients). Therefore, 667 patients with confirmed DENV infection were recruited in this study; 318 (47.7%) developed plasma leakage, whereas 349 (52.3%) had no plasma leakage (Fig 1). Only one (0.15%) patient expired due to DSS, severe bleeding, and multi-organ failure during this study; of 318 patients with plasma leakage, 14 (4.4%) patients developed DSS.

### Comparison of patients’ characteristics, dengue serotypes, clinical and laboratory findings between dengue patients with and without plasma leakage

Most patients’ characteristics were similar (Table 1), except for a significantly higher proportion of males among patients with plasma leakage than those without plasma leakage (P = 0.010). Similarly, patients with plasma leakage had significantly higher body mass index (BMI) (P = 0.009). Referral patients were found more among patients with plasma leakage than those without, which was significant (P = 0.006). However, dengue serotypes among patients with plasma leakage and those without were similar (P = 0.076).

**Table 1:** Patients’ characteristics, dengue serotypes, and outcomes of plasma leakage status (n = 667).

Characteristic	Total (n = 667)	With plasma leakage (n = 318)	Without plasma leakage (n = 349)	P-value
Fever onset (days)^a^	4.0 (3.0–5.0)	4.0 (3.0–5.0)	4.0 (3.0–5.0)	0.058
Age (years)^a^	26 (20–37)	27 (21–38)	25 (20–37)	0.091
Male^b^	348 (52.2)	183 (57.5)	165 (47.3)	0.010
BMI (kg/m^2^)^a^^,^^c^	23.0 (20.0–26.4)	23.2 (20.5–27.0)	22.7 (19.7–25.6)	0.009
Residence in Bangkok^b^	470 (70.5)	219 (68.9)	251 (71.9)	0.437
Referral patients^b^	308 (46.2)	165 (51.9)	143 (41.0)	0.006
Previous history of dengue^b^	108 (16.2)	53 (16.7)	55 (15.8)	0.832
Comorbid condition^b^	175 (26.2)	90 (28.3)	85 (24.4)	0.285
Hypertension^b^	34 (5.1)	20 (6.3)	14 (4.0)	0.246
Thalassemia or G6PD deficiency^b^	33 (4.9)	20 (6.3)	13 (3.7)	0.178
Hyperlipidemia^b^	29 (4.3)	14 (4.4)	15 (4.3)	1.000
Diabetes mellitus^b^	12 (1.8)	6 (1.9)	6 (1.7)	1.000
Peptic ulcer disease^b^	11 (1.6)	5 (1.6)	6 (1.7)	1.000
HBV or HCV infection^b^	7 (1.0)	3 (0.9)	4 (1.1)	1.000
HIV infection^b^	5 (0.7)	2 (0.6)	3 (0.9)	1.000
Asthma^b^	5 (0.7)	2 (0.6)	3 (0.9)	1.000
Confirmation test for dengue^b^				
Serotype 1	247 (37.0)	121 (38.1)	126 (36.1)	0.076
Serotype 2	283 (42.4)	145 (45.6)	138 (39.5)	
Serotype 3	44 (6.6)	16 (5.0)	28 (8.0)	
Serotype 4	93 (13.9)	36 (11.3)	57 (16.3)	
Hospitalization^b^	553 (82.9)	304 (95.6)	249 (71.3)	<0.001
Delay in hospitalization^b^	399 (59.8)	225 (70.8)	174 (49.9)	<0.001
Duration of hospitalization^a^^,^^d^ (days)	3.5 (2.4–4.6)	3.8 (2.8–4.8)	2.9 (2.0–3.9)	<0.001

BMI, body mass index; G6PD, glucose-6-phosphate dehydrogenase; HBV, hepatitis B virus; HCV, hepatitis C virus; HIV, human immunodeficiency virus.

^a^Data presented as median (interquartile range).

^b^Data presented as number (percentage).

^c^Body mass index was measured in 640 patients, of which 304 patients had plasma leakage, and 336 patients had no plasma leakage.

^d^In a total of 553 hospitalized patients, plasma leakage was observed in 204 patients, and plasma leakage did not occur in 249.

A significantly higher proportion of patients with plasma leakage were admitted in comparison to those without plasma leakage (P <0.001). In addition, a significant delay in hospitalization (P <0.001) and longer hospitalization duration (P <0.001) were more frequently observed among plasma leakage patients than those without plasma leakage (Table 1). Clinical symptoms and signs of patients were similar in both groups on days 1 to 2 of fever onset (Table 2). However, symptoms and signs of vomiting (P = 0.012), bleeding (P = 0.030), and hepatomegaly (P = 0.006) were observed significantly more frequently among patients with plasma leakage than those without plasma leakage on days 3 to 4 of fever onset (Table 2). Abdominal pain (P = 0.024), vomiting (P = 0.035), bleeding (P <0.001), and hepatomegaly (P <0.001) on days 5 to 6 of fever onset was observed in a significantly higher proportion of plasma leakage patients than patients without plasma leakage (Table 2). However, on days 7 to 8 of fever onset, retro-orbital pain was observed in a significantly lower percentage of plasma leakage patients (P = 0.032) while cough (P = 0.015), abdominal pain (P <0.001), bleeding (P <0.001), and hepatomegaly (P <0.001) were significantly higher (Table 2).

**Table 2:** Symptoms and signs of confirmed dengue by day of fever onset and plasma leakage status (n = 667).

Characteristic	Days 1 to 2 of fever onset				Days 3 to 4 of fever onset				Days 5 to 6 of fever onset				Days 7 to 8 of fever onset			
	Total (n = 123)	With PL (n = 59)	Without PL (n = 64)	P-value	Total (n = 453)	With PL (n = 231)	Without PL (n = 222)	P-value	Total (n = 624)	With PL (n = 312)	Without PL (n = 312)	P-value	Total (n = 558)	With PL (n = 283)	Without PL (n = 275)	P-value
Fever (%)	123 (100)	59 (100)	64 (100)	N/A	429 (94.7)	219 (94.8)	210 (94.6)	1.000	434 (69.6)	227 (72.8)	207 (66.3)	0.098	111 (19.9)	51 (18.0)	60 (21.8)	0.309
Myalgia (%)	111 (90.2)	51 (86.4)	60 (93.8)	0.289	392 (86.5)	204 (88.3)	188 (84.7)	0.321	420 (67.3)	209 (67.0)	211 (67.6)	0.932	165 (29.6)	78 (27.6)	87 (31.6)	0.336
Headache (%)	107 (87.0)	51 (86.4)	56 (87.5)	1.000	373 (82.3)	190 (82.3)	183 (82.4)	1.000	351 (56.3)	175 (56.1)	176 (56.4)	1.000	112 (20.1)	51 (18.0)	61 (22.2)	0.262
Chills (%)	96 (78.0)	45 (76.3)	51 (79.7)	0.811	293 (64.7)	149 (64.5)	144 (64.9)	1.000	226 (36.2)	112 (35.9)	114 (36.5)	0.934	20 (3.6)	6 (2.1)	14 (5.1)	0.097
Retro-orbital pain (%)	66 (53.7)	35 (59.3)	31 (48.4)	0.304	209 (46.1)	111 (48.1)	98 (44.1)	0.459	202 (32.4)	91 (29.2)	111 (35.6)	0.104	44 (7.9)	15 (5.3)	29 (10.5)	0.032
Nausea (%)	64 (52.0)	33 (55.9)	31 (48.4)	0.515	267 (58.9)	139 (60.2)	128 (57.7)	0.654	257 (41.2)	131 (42.0)	126 (40.4)	0.745	62 (11.1)	31 (11.0)	31 (11.3)	1.000
Cough (%)	29 (23.6)	13 (22.0)	16 (25.0)	0.861	147 (32.5)	77 (33.3)	70 (31.5)	0.757	217 (34.8)	119 (38.1)	98 (31.4)	0.093	140 (25.1)	84 (29.7)	56 (20.4)	0.015
Diarrhea (%)	26 (21.1)	17 (28.8)	9 (14.1)	0.075	140 (30.9)	78 (33.8)	62 (27.9)	0.214	138 (22.1)	76 (24.4)	62 (19.9)	0.210	54 (9.7)	30 (10.6)	24 (8.7)	0.545
Abdominal pain (%)	23 (18.7)	14 (23.7)	9 (14.1)	0.253	147 (32.5)	82 (35.5)	65 (29.3)	0.189	222 (35.6)	125 (40.1)	97 (31.1)	0.024	135 (24.2)	87 (30.7)	48 (17.5)	<0.001
Vomiting (%)	22 (17.9)	13 (22.0)	9 (14.1)	0.359	119 (26.3)	73 (31.6)	46 (20.7)	0.012	124 (19.9)	73 (23.4)	51 (16.3)	0.035	22 (3.9)	13 (4.6)	9 (3.3)	0.559
SOB (%)	18 (14.6)	9 (15.3)	9 (14.1)	1.000	75 (16.6)	37 (16.0)	38 (17.1)	0.851	83 (13.3)	43 (13.8)	40 (12.8)	0.814	36 (6.5)	14 (4.9)	22 (8.0)	0.195
Sore throat (%)	18 (14.6)	10 (16.9)	8 (12.5)	0.658	88 (19.4)	41 (17.7)	47 (21.2)	0.423	119 (19.1)	51 (16.3)	68 (21.8)	0.103	53 (9.5)	25 (8.8)	28 (10.2)	0.690
Sneezing (%)	17 (13.8)	9 (15.3)	8 (12.5)	0.857	55 (12.1)	26 (11.3)	29 (13.1)	0.656	53 (8.5)	22 (7.1)	31 (9.9)	0.251	19 (3.4)	13 (4.6)	6 (2.2)	0.181
Bleeding (%)	10 (8.1)	6 (10.2)	4 (6.2)	0.518^a^	98 (21.6)	60 (26.0)	38 (17.1)	0.030	216 (34.6)	134 (42.9)	82 (26.3)	<0.001	210 (37.6)	131 (46.3)	79 (28.7)	<0.001
Hepatomegaly (%)	2 (1.6)	1 (1.7)	1 (1.6)	1.000^a^	19 (4.2)	16 (6.9)	3 (1.4)	0.006	63 (10.1)	49 (15.7)	14 (4.5)	<0.001	51 (9.1)	43 (15.2)	8 (2.9)	<0.001
Rash (%)	2 (1.6)	2 (3.4)	0 (0)	0.228^a^	43 (9.5)	26 (11.3)	17 (7.7)	0.252	139 (22.3)	74 (23.7)	65 (20.8)	0.441	179 (32.1)	98 (34.6)	81 (29.5)	0.223

N/A, not applicable; PL, plasma leakage; SOB, shortness of breath.

^a^Data were analyzed using Fisher’s exact tests.

**Fig 2:**
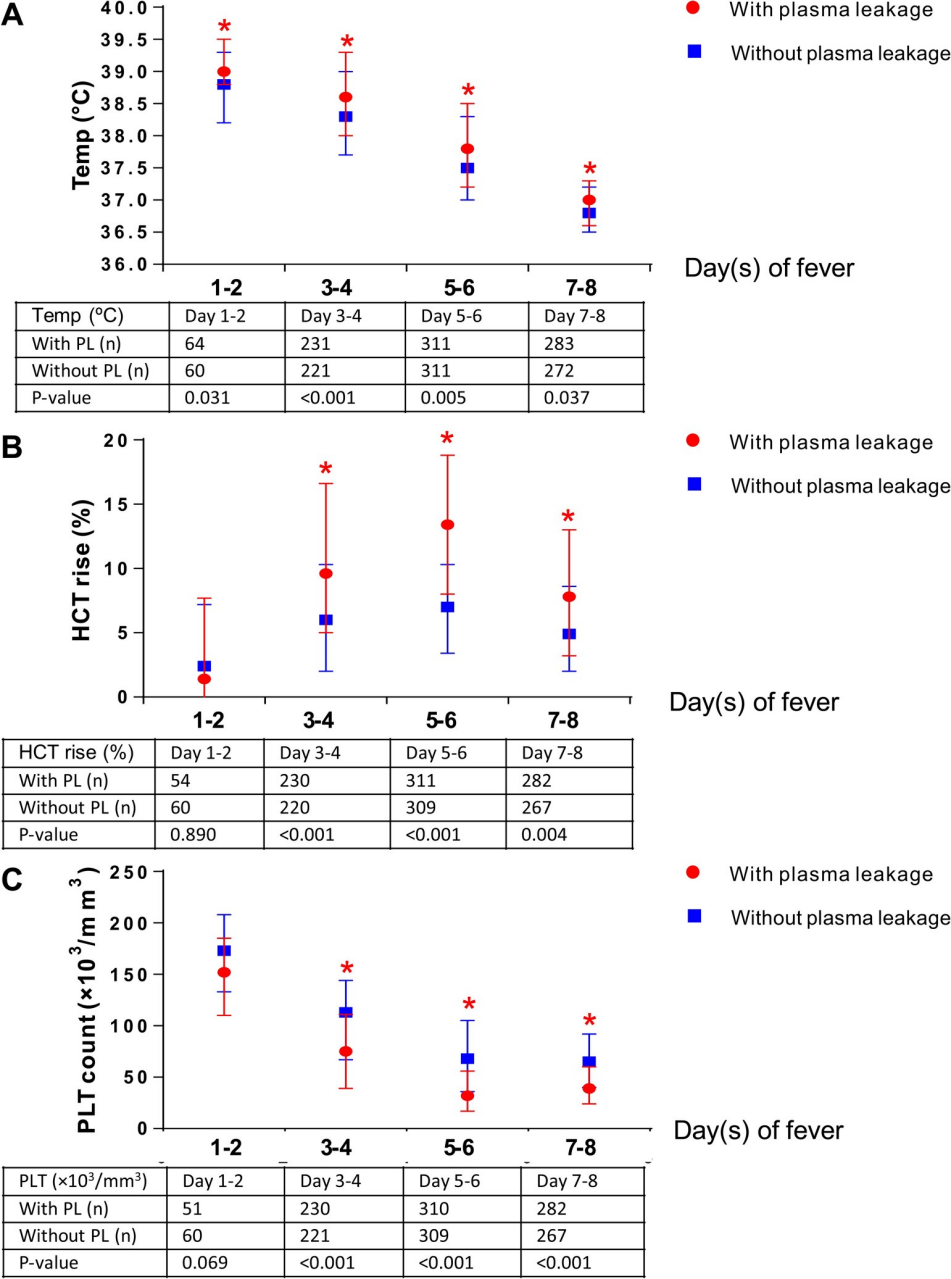
Changes in body temperature, hematocrit rise, and platelet count among confirmed dengue patients by combined day of fever onset. (a) The higest body temperature (°C) by combined day of fever onset among patients with and without plasma leakage. (b). The higest hematocrit rise (%) by combined day of fever onset among patients with and without plasma leakage. (c). The lowest platelet count (×10^3^/mm^3^) by combined day of fever onset among patients with and without plasma leakage. HCT, hematocrit; PL, plasma leakage; PLT, platelets; Temp, temperature.

Regarding vital signs and cumulative fluid balance (Tables 3 and 4), body temperature and mean arterial pressure (MAP) were similar between the two groups on days 1 to 2 of fever onset. Body temperature on days 3 to 5 and day 7 of fever onset were significantly higher among patients with plasma leakage than those without plasma leakage with P <0.05. When stratified by combined day of fever, body temperature (Fig 2A) between days 1 to 8 of fever onset remained significant (P <0.05). MAP on day 4 and days 6 to 8 of fever onset and cumulative fluid balance on days 5 to 8 of fever onset were significantly higher among patients with plasma leakage than those without plasma leakage with P <0.05 (Tables 3 and 4).

**Table 3:** Vital signs, cumulative fluid balance, and laboratory findings of confirmed dengue by days 1 to 4 of fever onset and plasma leakage status (n = 667).

Characteristic	Day 1 of fever onset				Day 2 of fever onset				Day 3 of fever onset				Day 4 of fever onset			
	Total	With PL	Without PL	P-value	Total	With PL	Without PL	P-value	Total	With PL	Without PL	P-value	Total	With PL	Without PL	P-value
Temp^a^ (°C)	n = 35	n = 17	n = 18		n = 99	n = 50	n = 49		n = 256	n = 131	n = 125		n = 419	n = 220	n = 199	
	39.0 (38.5–39.4)	39.0 (38.7–39.6)	38.7 (38.1–39.1)	0.134	38.9 (38.3–39.4)	39.0 (38.8–39.6)	38.8 (38.2–39.3)	0.066	38.5 (38.0–39.2)	38.7 (38.2–39.3)	38.3 (37.8–39.0)	<0.001	38.1 (37.5–38.8)	38.2 (37.8–39.0)	38.0 (37.8–38.6)	<0.001
MAP^a^ (mmHg)	n = 35	n = 17	n = 18		n = 98	n = 50	n = 48		n = 256	n = 131	n = 125		n = 419	n = 220	n = 199	
	83 (79–96)	83 (78–97)	84 (80–92)	0.568	84 (77–93)	86 (79–95)	80 (76–92)	0.062	84 (76–94)	85 (78–96)	83 (75–93)	0.061	84 (76–92)	86 (77–92)	82 (75–91)	0.036
Cumulative fluid^a^ (ml/day)	n = 4	n = 2	n = 2		n = 4	n = 2	n = 2		n = 123	n = 78	n = 45		n = 290	n = 184	n = 106	
	480 (119–765)	160 (78, 241)	750 (720, 780)	N/A	610 (396–1551)	610 (536, 683)	1095 (350, 1840)	N/A	500 (63–880)	540 (-42-922)	371 (130–708)	0.402	500 (-55-1105)	542 (-51-1238)	407 (-73- 966)	0.199
WBC^a^ (×10^3^ cells/mm^3^)	n = 27	n = 10	n = 17		n = 93	n = 48	n = 45		n = 256	n = 129	n = 127		n = 416	n = 218	n = 198	
	5.10 (4.10–7.00)	4.60 (4.00–6.48)	5.90 (4.00–7.70)	0.414	3.90 (2.90–5.50)	3.75 (2.65–5.10)	4.10 (3.35–5.65)	0.252	3.10 (2.48–4.20)	3.10 (2.55–4.10)	3.20 (2.40–4.20)	0.987	2.80 (2.30–3.80)	2.90 (2.30–3.82)	2.80 (2.20–3.72)	0.375
ALC^a^ (cells/mm^3^)	n = 19	n = 7	n = 12		n = 65	n = 34	n = 31		n = 196	n = 106	n = 90		n = 382	n = 198	n = 184	
	102 (41–141)	90 (45–108)	122 (0–176)	0.592	100 (32–202)	99 (0–207)	100 (44–207)	0.654	139 (76–224)	137 (76–212)	142 (76–236)	0.353	185 (92–331)	185 (93–390)	180 (89–306)	0.304
HCT rise^a^ (%)	n = 27	n = 10	n = 17		n = 93	n = 48	n = 45		n = 257	n = 130	n = 127		n = 416	n = 218	n = 198	
	0.25 (0–7.63)	0.29 (0–9.74)	0 (0–6.42)	0.711	2.88 (0–7.32)	1.58 (0–7.59)	3.31 (0–7.13)	0.911	5.22 (1.03–11.58)	6.36 (1.37–13.36)	4.50 (0.74–9.58)	0.040	6.73 (2.76–12.20)	8.43 (4.70–15.01)	5.11 (1.13–9.36)	<0.001
PLT count^a^ (×10^3^/mm^3^)	n = 27	n = 10	n = 17		n = 93	n = 48	n = 45		n = 257	n = 130	n = 127		n = 416	n = 218	n = 198	
	193 (154–215)	178 (148–215)	198 (158–214)	0.570	152 (116–181)	150 (110–172)	161 (126–207)	0.209	116 (75–146)	101 (64–142)	125 (90–156)	<0.001	85 (50–124)	73 (39–101)	106 (64–138)	<0.001
AST^a^ (U/l)	n = 27	n = 10	n = 17		n = 93	n = 48	n = 45		n = 153	n = 96	n = 57		n = 237	n = 141	n = 96	
	21 (17–27)	22 (18–29)	19 (16–22)	0.219	37 (28–54)	38 (30–61)	28 (24–41)	0.012	50 (34–90)	60 (38–118)	36 (24–63)	<0.001	80 (47–154)	97 (57–178)	58 (39–91)	<0.001
ALT^a^ (U/l)	n = 27	n = 10	n = 17		n = 93	n = 48	n = 45		n = 154	n = 97	n = 57		n = 237	n = 141	n = 96	
	22 (15–24)	22 (17–24)	20 (14–21)	0.125	30 (23–40)	32 (26–42)	23 (17–27)	<0.001	32 (21–56)	37 (25–76)	24 (16–37)	<0.001	51 (28–103)	54 (37–120)	38 (24–62)	<0.001
ALB^a^ (g/dl)	n = 24	n = 10	n = 14		n = 66	n = 48	n = 18		n = 85	n = 58	n = 27		n = 128	n = 92	n = 36	
	4.8 (4.5–5.0)	4.8 (4.6–5.0)	4.5 (4.3–5.2)	0.349	4.7 (4.5–4.9)	4.7 (4.5–4.9)	4.5 (4.3–4.6)	0.008	4.4 (4.2–4.7)	4.4 (4.2–4.7)	4.4 (4.3–4.9)	0.656	4.2 (3.9–4.5)	4.0 (3.8–4.5)	4.3 (4.2–4.6)	0.001

ALB, albumin; ALC, absolute lymphocyte count; ALT, alanine aminotransferase; AST, aspartate aminotransferase; HCT, hematocrit; MAP, mean arterial pressure; N/A, not applicable; PL, plasma leakage; PLT, platelets; Temp, temperature; WBC, white blood cell count.

^a^Data presented as median (interquartile range).

**Table 4:** Vital signs, cumulative fluid balance, and laboratory findings confirmed dengue patients by day 5 to 8 of fever onset and plasma leakage status (n = 667).

Characteristic	Day 5 of fever onset				Day 6 of fever onset				Day 7 of fever onset				Day 8 of fever onset			
	Total	With PL	Without PL	P-value	Total	With PL	Without PL	P-value	Total	With PL	Without PL	P-value	Total	With PL	Without PL	P-value
Temp^a^ (°C)	n = 554	n = 296	n = 258		n = 580	n = 300	n = 280		n = 553	n = 283	n = 270		n = 389	n = 222	n = 167	
	37.7 (37.0–38.4)	37.8 (37.2–38.5)	37.5 (37.0–38.3)	0.013	37.2 (36.8–37.8)	37.2 (36.8–37.8)	37.0 (36.7–37.8)	0.103	36.8 (36.5–37.2)	36.9 (36.6–37.8)	36.8 (36.5–37.1)	0.022	36.6 (36.5–37.0)	36.6 (36.5–37.0)	36.6 (36.5–37.0)	0.948
MAP^a^ (mmHg)	n = 554	n = 296	n = 258		n = 580	n = 300	n = 280		n = 554	n = 283	n = 271		n = 389	n = 222	n = 167	
	81 (74–90)	83 (75–91)	80 (74–88)	0.056	79 (72–87)	80 (73–87)	77 (71–85)	0.006	78 (72–86)	79 (73–88)	76 (71–84)	0.006	78 (72–87)	80 (73–89)	77 (71–83)	0.001
Cumulative fluid balance^a^ (ml/day)	n = 456	n = 276	n = 180		n = 521	n = 296	n = 225		n = 547	n = 305	n = 242		n = 548	n = 305	n = 243	
	580 (-12-1349)	670 (100–1645)	296 (-162- 966)	<0.001	630 (-192-1535)	915 (-36-1942)	291 (-482- 1005)	<0.001	381 (-565-1470)	600 (-362-1910)	232 (-653- 982)	<0.001	250 (-711-1385)	420 (-664-1682)	100 (-780- 936)	0.003
WBC^a^ (×10^3^ cells/mm^3^)	n = 548	n = 293	n = 255		n = 566	n = 294	n = 272		n = 544	n = 280	n = 264		n = 383	n = 221	n = 162	
	3.00 (2.30–4.30)	3.10 (2.30–4.60)	2.90 (2.30–3.90)	0.231	3.80 (2.70–5.32)	4.20 (2.70–5.90)	3.55 (2.70–4.90)	0.014	4.90 (3.50–6.60)	5.10 (3.90–6.80)	4.30 (3.22–6.28)	0.002	5.20 (4.20–6.70)	5.30 (4.40–6.90)	4.80 (3.88–6.40)	0.017
ALC^a^ (/mm^3^)	n = 517	n = 284	n = 233		n = 556	n = 292	n = 264		n = 540	n = 279	n = 261		n = 380	n = 219	n = 161	
	297 (160–692)	333 (180–797)	270 (144–599)	0.016	711 (336–1270)	818 (401–1362)	582 (266–1071)	<0.001	912 (500–1516)	943 (574–1496)	876 (458–1602)	0.239	869 (518–1334)	910 (525–1334)	816 (510–1348)	0.591
HCT rise^a^ (%)	n = 549	n = 294	n = 255		n = 567	n = 295	n = 272		n = 545	n = 281	n = 264		n = 384	n = 222	n = 162	
	7.61 (3.03–13.38)	10.59 (4.87–16.32)	5.50 (2.16–9.00)	<0.001	7.50 (3.01–12.27)	9.84 (4.52–16.11)	5.66 (1.70–9.09)	<0.001	5.22 (1.39–10.15)	6.84 (2.36–12.20)	3.78 (0.90–8.00)	<0.001	3.48 (0–7.69)	3.75 (0–9.06)	3.03 (0–6.94)	0.070
PLT count^a^ (×10^3^/mm^3^)	n = 548	n = 293	n = 255		n = 566	n = 294	n = 272		n = 544	n = 280	n = 264		n = 383	n = 221	n = 162	
	61 (31–97)	46 (23–76)	82 (47–119)	<0.001	45 (25–74)	34 (19–55)	62 (36–94)	<0.001	50 (29–78)	39 (24–60)	67 (39–73)	<0.001	64 (40–90)	57 (35–89)	72 (48–94)	<0.001
AST^a^ (U/l)	n = 269	n = 167	n = 102		n = 238	n = 152	n = 86		n = 179	n = 124	n = 55		n = 131	n = 95	n = 36	
	116 (58–212)	135 (79–270)	72 (42–132)	<0.001	128 (76–232)	154 (88–267)	107 (64–180)	0.002	141 (80–253)	152 (97–260)	89 (55–199)	0.003	142 (59–267)	165 (88–315)	72 (40–211)	0.004
ALT^a^ (U/l)	n = 269	n = 167	n = 102		n = 238	n = 152	n = 86		n = 179	n = 124	n = 55		n = 130	n = 95	n = 35	
	62 (34–126)	72 (45–127)	42 (23–91)	<0.001	77 (47–153)	88 (50–176)	62 (38–102)	0.001	99 (50–178)	108 (56–181)	70 (34–159)	0.016	117 (65–230)	135 (70–228)	98 (52–246)	0.166
ALB^a^ (g/dl)	n = 170	n = 125	n = 45		n = 165	n = 125	n = 40		n = 121	n = 98	n = 23		n = 75	n = 58	n = 17	
	4.0 (3.7–4.3)	3.9 (3.5–4.2)	4.3 (3.8–4.6)	<0.001	3.8 (3.4–4.1)	3.6 (3.3–4.0)	4.2 (4.0–4.6)	<0.001	3.7 (3.4–4.0)	3.6 (3.3–3.9)	4.2 (4.0–4.4)	<0.001	3.8 (3.4–4.2)	3.6 (3.4–4.0)	4.3 (4.2–4.8)	<0.001

ALB, albumin; ALC, absolute lymphocyte count; ALT, alanine aminotransferase; AST, aspartate aminotransferase; HCT, hematocrit; MAP, mean arterial pressure; PL, plasma leakage; PLT, platelets; Temp, temperature; WBC, white blood cell count.

^a^Data presented as median (interquartile range).

**Fig 3:**
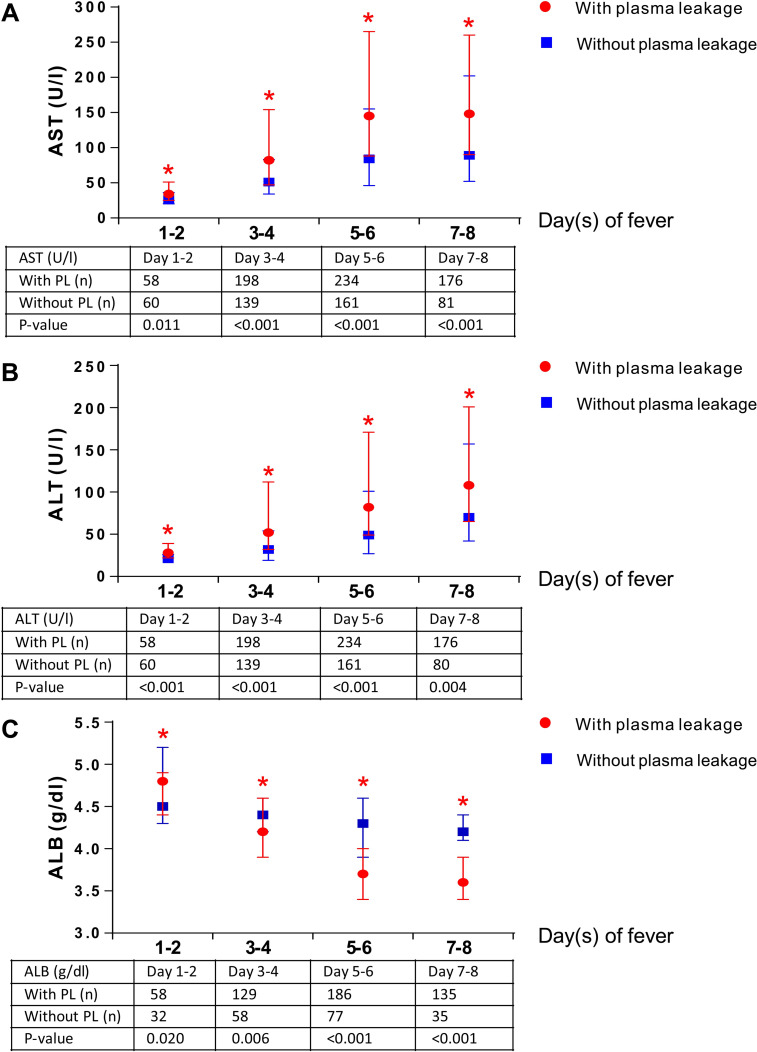
Changes in aspartate aminotransferase, alanine aminotransferase, and serum albumin levels among confirmed dengue patients by combined day of fever onset. (a) The highest level of AST (U/l) by combined day of fever onset among patients with and without plasma leakage. (b) The highest level of ALT (U/l) by combined day of fever onset among patients with and without plasma leakage. (c) The lowest level of serum ALB (g/dl) by combined day of fever onset among patients with and without plasma leakage. ALB, albumin; ALT, alanine aminotransferase; AST, aspartate aminotransferase; PL, plasma leakage.

Regarding laboratory findings (Tables 3 and 4), all laboratory findings including WBC, absolute lymphocyte count (ALC), HCT rise, PLT count, aspartate aminotransferase (AST), alanine aminotransferase (ALT), and serum ALB were similar on day 1 of fever onset. However, patients with plasma leakage had significantly higher WBC levels on days 6 to 8 of fever onset and higher ALC on days 5 to 6 of fever onset than those without plasma leakage (P <0.05). HCT rise (Tables 3 and 4) between days 3 to 7 was significantly higher among patients with plasma leakage (P <0.05). When stratified by combined day of fever, HCT rise (Fig 2B) between days 3 to 8 of fever onset was also significantly higher among patients with plasma leakage (P <0.05). However, PLT count (Tables 3 and 4) between days 3 to 8 of fever onset was significantly lower among patients with plasma leakage (P <0.001). When stratified by combined day of fever, PLT count (Fig 2C) between days 3 to 8 of fever was also significantly lower among plasma leakage patients (P <0.001). The levels of the liver enzymes, including AST and ALT (Tables 3 and 4) between days 2 to 8 of fever onset, when stratified by combined day of fever (Fig 3A and 3B) between days 1 to 8, were significantly higher among patients with plasma leakage than those without plasma leakage (P <0.05). Conversely, serum ALB levels (Tables 3 and 4) between days 4 to 8 of fever onset, when stratified by combined day of fever (Fig 3C) between days 3 to 8 were significantly lower among patients with plasma leakage than those without plasma leakage (P <0.05). However, serum ALB levels (Table 3) on day 2 of fever onset, when stratified by combined day of fever (Fig 3C) on days 1 to 2, were significantly higher among patients with plasma leakage than those without plasma leakage (P = 0.008 and P = 0.020, respectively).

### Univariate and multivariate analysis to predict the development of plasma leakage

Patients’ characteristics, clinical and laboratory findings associated with development of plasma leakage were then categorized (Table 5) and evaluated using univariate logistic regression analysis. The following factors were found to be associated with the development of plasma leakage including patients’ characteristics (male gender, BMI ≥25.0 kg/m^2^, and delay in hospitalization); the symptoms and signs on fever days 3 to 4 (vomiting, bleeding, hepatomegaly, and body temperature >38.5°C); the symptoms and signs on fever days 5 to 6 (abdominal pain); and the laboratory findings on days 3 to 4 of fever onset (HCT rise ≥10%, PLT count <100,000/mm^3^, and AST or ALT ≥100 U/l) (Table 6).

**Table 5:** Categorical data of patients’ characteristics, clinical, and laboratory findings included in logistic regression analysis.

Characteristics	Total	With plasma leakage	Without plasma leakage	P-value
*Patients’ characteristics*				
Gender (%)	n = 667	n = 318	n = 349	
Male	348 (52.2)	183 (57.5)	165 (47.3)	0.010
Female	319 (47.8)	135 (42.5)	184 (52.7)	
BMI (%)	n = 640	n = 304	n = 336	
≥25.0 kg/m^2^	211 (33.0)	114 (37.5)	97 (28.9)	0.025
<25.0 kg/m^2^	429 (67.0)	190 (62.5)	239 (71.1)	
Delay in hospitalization (%)	n = 667	n = 318	n = 349	
Yes	399 (59.8)	225 (70.8)	174 (49.9)	<0.001
No	268 (40.2)	93 (29.2)	175 (50.1)	
*Symptoms and signs on fever days 3 to 4*				
Vomiting (%)	n = 453	n = 231	n = 222	
Yes	119 (26.3)	73 (31.6)	46 (20.7)	0.012
No	334 (73.7)	158 (68.4)	176 (79.3)	
Bleeding (%)	n = 453	n = 231	n = 222	
Yes	98 (21.6)	60 (26.0)	38 (17.1)	0.030
No	355 (78.4)	171 (74.0)	184 (82.9)	
Hepatomegaly (%)	n = 453	n = 231	n = 222	
Yes	19 (4.2)	16 (6.9)	3 (1.4)	0.006
No	434 (95.8)	215 (93.1)	219 (98.6)	
Temp (%)	n = 452	n = 231	n = 221	
>38.5°C	201 (44.5)	117 (50.6)	84 (38.0)	0.009
≤38.5°C	251 (55.5)	114 (49.4)	137 (62.0)	
*Symptoms and signs on fever days 5 to 6*				
Abdominal pain (%)	n = 624	n = 312	n = 312	
Yes	222 (35.6)	125 (40.1)	97 (31.1)	0.024
No	402 (64.4)	187 (59.9)	215 (68.9)	
*Laboratory findings on fever days 3 to 4*				
HCT rise (%)	n = 451	n = 230	n = 221	
≥10%	181 (40.1)	116 (50.4)	65 (29.4)	<0.001
<10%	270 (59.9)	114 (49.6)	156 (70.6)	
PLT count (%)	n = 451	n = 230	n = 221	
<100,000/mm^3^	252 (55.9)	163 (70.9)	89 (40.3)	<0.001
≥100,000/mm^3^	199 (44.1)	67 (29.1)	132 (59.7)	
AST or ALT (%)	n = 337	n = 198	n = 139	
≥100 U/l	75 (22.3)	60 (30.3)	15 (10.8)	<0.001
<100 U/l	262 (77.7)	138 (69.7)	124 (89.2)	

ALT, alanine aminotransferase; AST, aspartate aminotransferase; BMI, body mass index; HCT, hematocrit; PLT, platelets; Temp, temperature.

**Table 6:** Univariate and multivariate logistic regression to determine independent risk factors associated with plasma leakage among dengue patients.

Characteristics	Univariate logistic regression analysis			Multivariate logistic regression analysis		
	n	OR (95% CI)	P-value	n	OR (95% CI)	P-value
Gender	667					
Male		1.512 (1.113–2.053)	0.008			
Female		Reference				
BMI	640			293		
≥ 25.0 kg/m^2^		1.478 (1.062–2.058)	0.021		1.784 (1.040–3.057)	0.035
< 25.0 kg/m^2^		Reference			Reference	
Delay in hospitalization	667					
Yes		2.433 (1.767–3.351)	<0.001			
No		Reference				
Vomiting	453					
Yes		1.768 (1.153–2.709)	0.009			
No		Reference				
Bleeding	453					
Yes		1.699 (1.076–2.682)	0.023			
No		Reference				
Hepatomegaly	453			293		
Yes		5.433 (1.561–13.912)	0.008		4.042 (0.857–19.055)	0.078
No		Reference			Reference	
Abdominal pain	453					
Yes		1.482 (1.065–2.060)	0.019			
No		Reference				
Temp	452					
>38.5°C		1.674 (1.151–2.434)	0.007			
≤38.5°C		Reference				
HCT rise	451					
≥10%		2.442 (1.657–3.600)	<0.001			
<10%		Reference				
PLT count	451			293		
<100,000/mm^3^		3.608 (2.440–5.337)	<0.001		2.151 (1.269–3.647)	0.004
≥100,000/mm^3^		Reference			Reference	
AST or ALT	337			293		
≥100 U/l		3.202 (1.921–5.339)	<0.001		2.189 (1.231–3.891)	0.008
<100 U/l		Reference			Reference	

ALT, alanine aminotransferase; AST, aspartate aminotransferase; BMI, body mass index; CI, confidence interval; HCT, hematocrit; OR, odds ratio; PLT, platelets; Temp, temperature.

The independent factors associated with plasma leakage identified by stepwise multiple logistic regression analysis were BMI ≥25.0 kg/m^2^ (odds ratio [OR] = 1.784; 95% CI = 1.040–3.057; P = 0.035), PLT count <100,000/mm^3^ on days 3 to 4 of fever onset (OR = 2.151; 95% CI = 1.269–3.647; P = 0.004), and AST or ALT ≥100 U/l on days 3 to 4 of fever onset (OR = 2.189; 95% CI = 1.231–3.891; P = 0.008) (Table 6).

### Plasma-leak score for predicting plasma leakage

**Fig 4:**
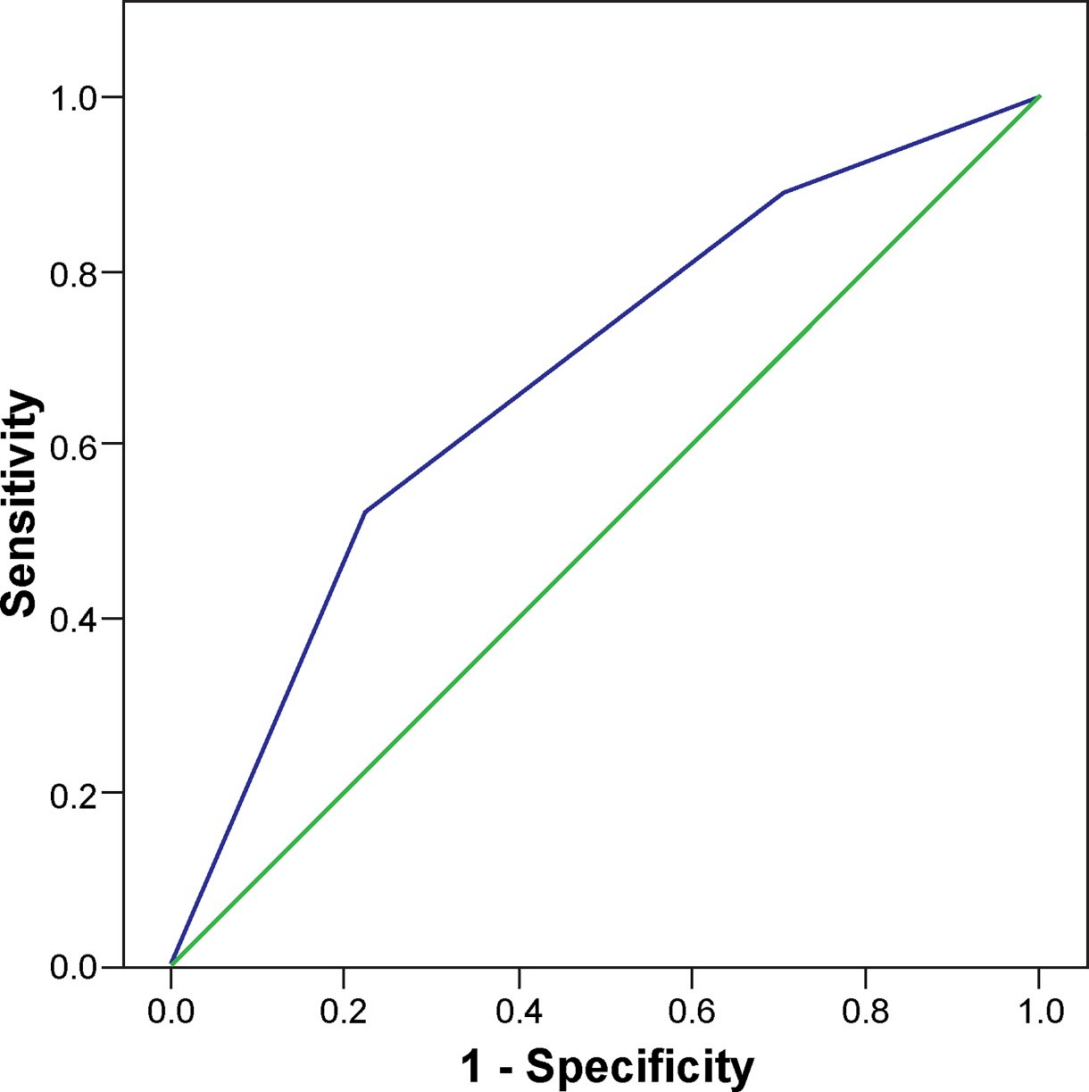
Receiver operating characteristic curve of the plasma-leak score for predicting plasma leakage among dengue patients.

Three factors, including BMI ≥25.0 kg/m^2^, PLT count <100,000/mm^3^, and AST or ALT ≥100 U/l were used to develop a score for predicting plasma leakage called a plasma-leak score. Because these three parameters had evidence of equality, each of the three independent factors were weighted to give a score of 1 with a total score of 3. A combined score was then evaluated using a logistic regression model to forecast the capacity for predicting the occurrence of plasma leakage. Higher scores were associated with increased occurrence of plasma leakage, with ORs of 2.017 (95% CI = 1.052–3.869; P = 0.035), 6.158 (95% CI = 2.914–13.015; P <0.001) and 6.300 (95% CI = 2.419–16.407; P <0.001) for a combined score of 1, 2, and 3, respectively (Table 7). The AUROC for predicting plasma leakage was 0.677 (95% CI = 0.616–0.739) (Fig 4).

**Table 7:** Multivariate logistic regression of a combined plasma-leak score for predicting plasma leakage.

Score	n	Odds ratio (95% CI)	P-value
0	56	1.00 (Reference)	N/A
1	123	2.017 (1.052–3.869)	0.035
2	84	6.158 (2.914–13.015)	<0.001
3	36	6.300 (2.419–16.407)	<0.001

CI, confidence interval; N/A, not applicable.

### Prognostic values of plasma-leak score for identifying plasma leakage

The prognostic values of the plasma-leak score for identifying plasma leakage are summarized in Table 8. The sensitivity was 88.8% (95% CI = 83.2–93.0%) for a score ≥1. The specificities increased to 77.7% (95% CI = 69.2–84.8%) and 93.4% (95% CI = 87.4–97.1%) for a score of ≥2 and 3, respectively. Similarly, the PPVs increased to 77.5% (95% CI = 70.6–83.2%) and 77.8% (95% CI = 62.3–88.1%) for a score of ≥2 and 3, respectively.

**Table 8:** Prognostic values of the plasma-leak score for predicting plasma leakage.

Total score	With plasma leakage (n = 178)	Without plasma leakage (n = 121)	Sensitivity (95% CI)	Specificity (95% CI)	PPV (95% CI)	NPV (95% CI)	LR+ (95% CI)	LR- (95% CI)
≥1	158	85	88.8 (83.2–93.0)	29.8 (21.8–38.7)	65.0 (62.1–67.9)	64.3 (52.3–74.7)	1.3 (1.1–1.4)	0.4 (0.2–0.6)
≥2	93	27	52.2 (44.6–59.8)	77.7 (69.2–84.8)	77.5 (70.6–83.2)	52.5 (48.0–57.0)	2.3 (1.6–3.4)	0.6 (0.5–0.7)
3	28	8	15.7 (10.7–21.9)	93.4 (87.4–97.1)	77.8 (62.3–88.1)	43.0 (41.0–44.9)	2.4 (1.1–5.0)	0.9 (0.8–1.0)

CI, confidence interval; LR+, likelihood ratio positive; LR-, likelihood ratio negative; NPV, negative predictive value; PPV, positive predictive value.

## Discussion

This prospective observational study was conducted at the Hospital for Tropical Diseases in Bangkok, Thailand, among dengue patients aged ≥15 years between March 2018 and February 2020 to determine predictors and a predictive score for plasma leakage. The median age of 26 years in the patients is similar to that of a previous report with a mean age of 30 years in adults affected by dengue [28]. The median time of the first visit to the hospital was 4 days of fever onset, similar to previous studies [8,16,26]. Differences in age and comorbid diseases were not observed between the two groups. However, previous studies have reported that diabetes, hypertension, cardiac disorders, and asthma patients were at increased risk for severe manifestations of dengue [9–12,29]. In our study, most clinical and laboratory findings were significantly different between plasma leakage patients and those without plasma leakage starting on days 3 to 4 of fever onset. Bleeding was the most common condition associated with plasma leakage observed on days 5 to 8 of fever onset in this study.

Plasma leakage and bleeding are the hallmark of dengue hemorrhagic fever (DHF) and are associated with death from dengue [5,7]. This might be due to the linkage on the disease’s pathogenesis via cytokine storm and antibodies response on the vascular endothelial cells and the hemostatic abnormalities after DENV infection [30,31]. Our study also showed that MAP and fluid balance were significantly higher among patients with plasma leakage. This might be because patients with plasma leakage received more fluid replacement than those without plasma leakage [5]. Currently, plasma leakage is the main pathophysiological hallmark of DHF [28]. According to the modified WHO/SEARO 2011 criteria, plasma leakage is the major criteria for distinguishing DHF from dengue fever (DF), without the necessity of bleeding [5,6]. Early recognition of plasma leakage can lead to appropriate fluid administration and then prevent the development of DSS, which is the most common cause of death from dengue [3–7]. However, most previous studies regarding plasma leakage predictors have varied results due to differences in study design, participant composition, and case definition of severe manifestations of dengue [8–19].

This study identified three independent factors associated with plasma leakage. They were BMI ≥25.0 kg/m^2^, PLT count <100,000/mm^3^ on days 3 to 4 of fever onset, and AST or ALT ≥100 U/l on days 3 to 4 of fever onset. As per systematic review and meta-analysis, most studies on the association of BMI and dengue severity were conducted in children. They showed that obese children had a higher risk for developing DHF or severe dengue compared to non-obese children; this is due to the stronger immune response in obese children than undernourished or normal weighted children [32,33]. A retrospective study from Malaysia showed that adult dengue patients with BMI ≥27.5 kg/m^2^ were at risk for elevated ALT, creatinine level, raised HCT, the occurrence of chills and rigors, high body temperature, and high systolic blood pressure [34].

The possible pathophysiological mechanisms for developing plasma leakage in obese patients with dengue might be due to endothelial dysfunction caused by the chronic release of pro-inflammatory cytokines from elevated leptin levels and production of reactive oxygen species (ROS). These would precipitate endothelial damage in addition to cytokine storm after DENV infection. Moreover, downregulation of AMP-protein kinase in obese patients could lead to lipid accumulation in the endoplasmic reticulum, facilitating viral replication [31,34,35].

We found significantly higher serum ALB levels among patients with plasma leakage than those without plasma leakage on days 1 to 2 of fever onset. This might be due to the well-nourished status of plasma leakage patients, which might build up a stronger immune response. However, serum ALB levels of patients with plasma leakage decreased on days 3 to 8 of fever onset and was significantly lower than those without plasma leakage. These might be due to the leakage of serum ALB into the extravascular compartment from a cytokine-mediated increase in vascular permeability by endothelial glycocalyx damage, which is the primary pathogenesis of plasma leakage [5,30,31]. In 2021, a systematic review and meta-analysis identifying risk predictors of progression to severe disease, defined as severe dengue or DHF during the febrile phase of dengue, was published. The authors showed that all included studies in the analysis consistently reported that patients who progressed to DHF had lower serum ALB levels than those who did not progress to DHF [36].

On days 3 to 4 of fever onset, PLT count <100,000/mm^3^ was a predictor for plasma leakage similar to a multicenter retrospective study in Thailand [15]. Plasma leakage and thrombocytopenia has a link with the pathogenesis of the disease via cytokine storm and cross-reactive immunoglobulin M type of antibodies after DENV infection, which is the potential mechanism of vascular pathology and PLT destruction [31,37,38].

In addition, AST or ALT ≥100 U/l on days 3 to 4 of fever onset was also a predictor for plasma leakage. Similarly, previous reports showed that elevated transaminases were an independent factor associated with severe manifestations of dengue [12,13]. Hepatocytes and Kupffer cells in the liver are important targets of DENV, which results in direct damage of liver cells by apoptosis and release of pro-inflammatory cytokines, which also results in endothelial damage [39,40]. In our study, the dosage of acetaminophen recommended by the National Thai guidelines, which relies on the US Food and Drug Administration suggestion for the reduction of fever, was used. The maximum daily dose of acetaminophen for an adult is 3000 mg with a recommended dosage of 500 mg every 6 hours. In 2021, a systematic review and meta-analysis identifying risk predictors of progression to severe disease, defined as severe dengue or DHF during the febrile phase of dengue, showed that all included studies in the analysis consistently reported that higher levels of AST or ALT were associated with progression to severe disease [36]. Thus, it is suggested that the elevation of AST or ALT in our study accounted for the association with DENV infection rather than with acetaminophen-induced hepatoxicity.

Till date, few studies developed a predictive score for dengue severity in adults, including the dengue score for predicting pleural effusion and/or ascites [13] and the clinical risk score for prediction of severe dengue [41]. In our study, a plasma-leak score was developed for identifying plasma leakage using a score as 1 for each parameter, including BMI ≥25.0 kg/m^2^, PLT count <100,000/mm^3^ on fever days 3 to 4, and AST or ALT ≥100 U/l on fever days 3 to 4. The plasma-leak score had a good discriminative ability with AUROC of 0.677. The sensitivity for the occurrence of plasma leakage was 88.8% for a score ≥1. The specificity for the occurrence of plasma leakage rose to 77.7% score ≥2, and as high as 93.4% for 3. The PPV was also increased to 77.5% for a score ≥2 and 77.8% for 3. These predictors are simple routine parameters for the early identification of patients who are at risk for plasma leakage, and the plasma-leak score could help with risk stratification of dengue. The risk stratification could help physicians to provide close observation as well as early and appropriate management, to prevent the progression to DSS. Ultimately, these measures would help in reducing the hospital cost, cost to the patients, and healthcare personnel workload.

However, this study’s limitations were that the study was conducted in a single-center, which is the referral center for tropical diseases in Bangkok, Thailand, and the plasma-leak score’s external validity needs to be evaluated.

## Conclusions

Dengue patients with BMI ≥25.0 kg/m^2^ or who presented with PLT count <100,000/mm^3^, or AST or ALT ≥100 U/L on days 3 to 4 of fever onset are at risk for the occurrence of plasma leakage. Patients with a plasma-leak score ≥1 had high sensitivity (88.8%) for the development of plasma leakage, and those with a plasma-leak score of 3 had high specificity (93.4%) for plasma leakage. This simple and easily accessible clinical score might help physicians provide close observation with early and appropriate clinical management of dengue even in resource-limited settings.

## References

[1] Bhatt S, Gething PW, Brady OJ, Messina JP, Farlow AW, Moyes CL, et al. The global distribution and burden of dengue. Nature. 2013;496(7446): 504–507. doi: 10.1038/nature12060 . 23563266 PMC3651993

[2] Chumpu R, Khamsemanan N, Nattee C. The association between dengue incidences and provincial-level weather variables in Thailand from 2001 to 2014. PLOS ONE. 2019;14(12): e0226945. doi: 10.1371/journal.pone.0226945 . 31877191 PMC6932763

[3] Woon YL, Hor CP, Hussin N, Zakaria A, Goh PP, Cheah WK. A two-year review on epidemiology and clinical characteristics of dengue deaths in Malaysia, 2013–2014. PLOS Negl Trop Dis. 2016;10(5): e0004575. doi: 10.1371/journal.pntd.0004575 . 27203726 PMC4874788

[4] World Health Organization (WHO). Global strategy for dengue prevention and control 2012–2020. Geneva: WHO; 2012. Available from: http://www.who.int/denguecontrol/9789241504034/en/. [Accessed 07 August 2017].

[5] Kalayanarooj S. Clinical manifestations and management of dengue/DHF/DSS. Trop Med Health. 2011;39(Suppl 4): 83–87. doi: 10.2149/tmh.2011-S10 . 22500140 PMC3317599

[6] World Health Organization (WHO). Comprehensive guidelines for prevention and control of dengue and dengue hemorrhagic fever. Revised and expanded edition. New Delhi, India: WHO; 2011. Available from: http://www.searo.who.int/entity/vector_borne_tropical_diseases/documents/SEAROTPS60/en/. [Accessed 07 August 2017].

[7] da Silva NS, Undurraga EA, da Silva Ferreira ER, Estofolete CF, Nogueira ML. Clinical, laboratory, and demographic determinants of hospitalization due to dengue in 7613 patients: A retrospective study based on hierarchical models. Acta Trop. 2018;177: 25–31. doi: 10.1016/j.actatropica.2017.09.025 . 28964768

[8] Thomas L, Brouste Y, Najioullah F, Hochedez P, Hatchuel Y, Moravie V, et al. Predictors of severe manifestations in a cohort of adult dengue patients. J Clin Virol. 2010;48(2): 96–99. doi: 10.1016/j.jcv.2010.03.008 . 20362495

[9] Mallhi TH, Khan AH, Adnan AS, Sarriff A, Khan YH, Jummaat F. Clinico-laboratory spectrum of dengue viral infection and risk factors associated with dengue hemorrhagic fever: a retrospective study. BMC Infect Dis. 2015;15: 399. doi: 10.1186/s12879-015-1141-3 . 26423145 PMC4590689

[10] Lee IK, Hsieh CJ, Lee CT, Liu JW. Diabetic patients suffering dengue are at risk for development of dengue shock syndrome/severe dengue: Emphasizing the impacts of co-existing comorbidity(ies) and glycemic control on dengue severity. J Microbiol Immunol Infect. 2020;53(1): 69–78. doi: 10.1016/j.jmii.2017.12.005 . 30146413

[11] Pang J, Salim A, Lee VJ, Hibberd ML, Chia KS, Leo YS, et al. Diabetes with hypertension as risk factors for adult dengue hemorrhagic fever in a predominantly dengue serotype 2 epidemic: a case control study. PLOS Negl Trop Dis. 2012;6(5): e1641. doi: 10.1371/journal.pntd.0001641 . 22563519 PMC3341340

[12] Aung KL, Thanachartwet V, Desakorn V, Chamnanchanunt S, Sahassananda D, Chierakul W, et al. Factors associated with severe clinical manifestation of dengue among adults in Thailand. Southeast Asian J Trop Med Public Health. 2013;44(4): 602–612. . 24050093

[13] Suwarto S, Nainggolan L, Sinto R, Effendi B, Ibrahim E, Suryamin M, et al. Dengue score: a proposed diagnostic predictor for pleural effusion and/or ascites in adults with dengue infection. BMC Infect Dis. 2016;16: 322. doi: 10.1186/s12879-016-1671-3 . 27391122 PMC4938904

[14] Tee HP, How SH, Jamalludin AR, Safhan MN, Sapian MM, Kuan YC, et al. Risk factors associated with development of dengue haemorrhagic fever or dengue shock syndrome in adults in Hospital Tengku Ampuan Afzan Kuantan. Med J Malays. 2009;64(4): 316–320. . 20954558

[15] Temprasertrudee S, Thanachartwet V, Desakorn V, Keatkla J, Chantratita W, Kiertiburanakul S. A multicenter study of clinical presentation and predictive factors for severe manifestation of dengue in adults. Open Forum Infect Dis. 2016;3 suppl 1(s1): 592. doi: 10.1093/ofid/ofw172.455 29709965

[16] Thanachartwet V, Desakorn V, Sahassananda D, Jittmittraphap A, Oer-areemitr N, Osothsomboon S, et al. Serum procalcitonin and peripheral venous lactate for predicting dengue shock and/or organ failure: a prospective observational study. PLOS Negl Trop. 2016;10(8). doi: 10.1371/journal.pntd.0004961 . 27564863 PMC5001649

[17] Bur R, Suwarto S, Santoso WD, Harimurti K. Serum lactate as predictor and diagnostic biomarker of plasma leakage in adult dengue patients. UnivMed. 2016;35(3): 213–221. doi: 10.18051/UnivMed.2016.v35.213–221

[18] Tissera H, Rathore APS, Leong WY, Pike BL, Warkentien TE, Farouk FS, et al. Chymase level is a predictive biomarker of dengue hemorrhagic fever in pediatric and adult patients. J Infect Dis. 2017;216(9): 1112–1121. doi: 10.1093/infdis/jix447 . 28968807 PMC5853622

[19] Bozza FA, Cruz OG, Zagne SM, Azeredo EL, Nogueira RM, Assis EF, et al. Multiplex cytokine profile from dengue patients: MIP-1beta and IFN-gamma as predictive factors for severity. BMC Infect Dis. 2008;8: 86. doi: 10.1186/1471-2334-8-86 . 18578883 PMC2474613

[20] von Elm E, Altman DG, Egger M, Pocock SJ, Gøtzsche PC, Vandenbroucke JP, et al. Strengthening the Reporting of Observational Studies in Epidemiology (STROBE) statement: guidelines for reporting observational studies. BMJ. 2007;335: 806–808. doi: 10.1136/bmj.39335.541782.AD . 17947786 PMC2034723

[21] Korevaar DA, van Enst WA, Spijker R, Bossuyt PM, Hooft L. Reporting quality of diagnostic accuracy studies: a systematic review and meta-analysis of investigations on adherence to STARD. Evid Based Med. 2014;19: 47–54. doi: 10.1136/eb-2013-101637 . 24368333

[22] Lanciotti RS, Calisher CH, Gubler DJ, Chang GJ, Vorndam AV. Rapid detection and typing of dengue viruses from clinical samples by using reverse transcriptase-polymerase chain reaction. J Clin Microbiol. 1992;30: 545–551. doi: 10.1128/jcm.30.3.545-551.1992 . 1372617 PMC265106

[23] Reynes JM, Ong S, Mey C, Ngan C, Hoyer S, Sall AA. Improved molecular detection of dengue virus serotype 1 variants. J Clin Microbiol. 2003;41: 3864–3867. doi: 10.1128/JCM.41.8.3864-3867.2003 . 12904404 PMC179838

[24] Vorndam V, Beltran M. Enzyme-linked immunosorbent assay-format microneutralization test for dengue viruses. Am J Trop Med Hyg. 2002;66: 208–212. doi: 10.4269/ajtmh.2002.66.208 . 12135295

[25] Putnak JR, de la Barrera R, Burgess T, Pardo J, Dessy F, Gheysen D, et al. Comparative evaluation of three assays for measurement of dengue virus neutralizing antibodies. Am J Trop Med Hyg. 2008;79: 115–122. doi: 10.4269/ajtmh.2008.79.115 . 18606774

[26] Tantawichien T. Dengue fever and dengue haemorrhagic fever in adolescents and adults. Paediatr Int Child Health. 2012;32(Suppl 1): 22–27. doi: 10.1179/2046904712Z.00000000049 . 22668446 PMC3381442

[27] Gibot S, Béné MC, Noel R, Massin F, Guy J, Cravoisy A, et al. Combination biomarkers to diagnose sepsis in the critically ill patient. Am J Respir Crit Care Med. 2012;186: 65–71. doi: 10.1164/rccm.201201-0037OC . 22538802

[28] Guo C, Zhou Z, Wen Z, Liu Y, Zeng C, Xiao D, et al. Global epidemiology of dengue outbreaks in 1990–2015: a systematic review and meta-analysis. Front Cell Infect Microbiol. 2017;7: 317. doi: 10.3389/fcimb.2017.00317 . 28748176 PMC5506197

[29] Pang J, Hsu JP, Yeo TW, Leo YS, Lye DC. Diabetes, cardiac disorders and asthma as risk factors for severe organ involvement among adult dengue patients: A matched case-control study. Sci Rep. 2017:7: 39872. doi: 10.1038/srep39872 . 28045096 PMC5206669

[30] Wang WH, Urbina AN, Chang MR, Assavalapsakul W, Lu PL, Chen YH, et al. Dengue hemorrhagic fever—A systemic literature review of current perspectives on pathogenesis, prevention and control. J Microbiol Immunol Infect. 2020;53(6): 963–978. doi: 10.1016/j.jmii.2020.03.007 . 32265181

[31] St John AL, Abraham SN, Gubler DJ. Barriers to preclinical investigations of anti-dengue immunity and dengue pathogenesis. Nat Rev Microbiol. 2013;11(6): 420–426. doi: 10.1038/nrmicro3030 . 23652323

[32] Trang NTH, Long NP, Hue TTM, Hung LP, Trung TD, Dinh DN, et al. Association between nutritional status and dengue infection: a systematic review and meta-analysis. BMC Infect Dis. 2016;16: 172. doi: 10.1186/s12879-016-1498-y . 27097934 PMC4839161

[33] Zulkipli MS, Dahlui M, Jamil N, Peramalah D, Wai HVC, Bulgiba A, et al. The association between obesity and dengue severity among pediatric patients: A systematic review and meta-analysis. PLOS Negl Trop Dis. 2018;12(2): e0006263. doi: 10.1371/journal.pntd.0006263 . 29415036 PMC5819989

[34] Tan VPK, Ngim CF, Lee EZ, Ramadas A, Pong LY, Ng JI, et al. The association between obesity and dengue virus (DENV) infection in hospitalised patients. PLOS ONE. 2018;13(7): e0200698. doi: 10.1371/journal.pone.0200698 . 30016369 PMC6049924

[35] Gallagher P, Chan KR, Rivino L, Yacoub S. The association of obesity and severe dengue: possible pathophysiological mechanisms. J Infectol. 2020;81: 10–16. doi: 10.1016/j.jinf.2020.04.039 . 32413364

[36] Sangkaew S, Ming D, Boonyasiri A, Honeyford K, Kalayanarooj S, Yacoub S, et al. Risk predictors of progression to severe disease during the febrile phase of dengue: a systematic review and meta-analysis. Lancet Infect Dis. 2021: S1473-3099(20)30601-0. doi: 10.1016/S1473-3099(20)30601-0 . 33640077 PMC8240557

[37] Lin CF, Lei HY, Liu CC, Liu HS, Yeh TM, Wang ST, et al. Generation of IgM anti-platelet autoantibody in dengue patients. J Med Virol. 2001;63(2): 143–149. doi: 10.1002/1096-9071(20000201)63:2&lt;143::AID-JMV1009&gt;3.0.CO;2-L . 11170051

[38] Soundravally R, Sankar P, Bobby Z, Hoti SL. Oxidative stress in severe dengue viral infection: association of thrombocytopenia with lipid peroxidation. Platelets. 2008;19(6): 447–454. doi: 10.1080/09537100802155284 . 18925513

[39] Couvelard A, Marianneau P, Bedel C, Drouet MT, Vachon F, Hénin D, et al. Report of a fatal case of dengue infection with hepatitis: demonstration of dengue antigens in hepatocytes and liver apoptosis. Hum Pathol. 1999;30(9): 1106–1110. doi: 10.1016/s0046-8177(99)90230-7 . 10492047

[40] Marianneau P, Steffan AM, Royer C, Drouet MT, Jaeck D, Kirn A, et al. Infection of primary cultures of human Kupffer cells by Dengue virus: no viral progeny synthesis, but cytokine production is evident. J Virol. 1999;73(6): 5201–5206. doi: 10.1128/JVI.73.6.5201-5206.1999 . 10233989 PMC112571

[41] Lee IK, Liu JW, Chen YH, Chen YC, Tsai CY, Huang SY, et al. Development of a simple clinical risk score for early prediction of severe dengue in adult patients. PLOS ONE. 2016;11(5): e0154772. doi: 10.1371/journal.pone.0154772 . 27138448 PMC4854400

